# Phase 1 combination study of Eribulin mesylate with trastuzumab for advanced or recurrent human epidermal growth factor receptor 2 positive breast cancer

**DOI:** 10.1007/s10637-014-0161-y

**Published:** 2014-09-23

**Authors:** Hirofumi Mukai, Toshiaki Saeki, Ken Shimada, Yoichi Naito, Nobuaki Matsubara, Tadashi Nakanishi, Hiroshi Obaishi, Masayuki Namiki, Yasutsuna Sasaki

**Affiliations:** 1 Department of Breast and Medical Oncology, National Cancer Center Hospital East, 6-5-1 Kashiwanoha, Kashiwa, Chiba 277-8577 Japan; 2 Department of Breast Oncology Cancer Center, International Medical Center Saitama Medical University, 1397-1 Yamane, Hidaka, Saitama 350-1298 Japan; 3 Department of Internal Medicine, Showa University Northern Yokohama Hospital, 35-1 Chigasaki-chuo, Tsuzuki-ku, Yokohama, Kanagawa 224-8503 Japan; 4 Eisai Co., Ltd, 4-6-10 Koishikawa, Bunkyo-ku, Tokyo, 112-8088 Japan; 5 Division of Medical Oncology-Department of Medicine, Showa University School of Medicine, 1-5-8 Hatanodai, Shinagawa-ku, Tokyo, 142-8666 Japan

**Keywords:** Eribulin mesylate, HER2 positive breast cancer, Japanese patients, Phase 1 study, Trastuzumab

## Abstract

Eribulin mesylate (Halaven®) is a novel inhibitor of microtubule dynamics that has demonstrated a survival benefit in patients with locally recurrent or metastatic breast cancer who previously received at least two chemotherapeutic regimens including an anthracycline and a taxane. Although trastuzumab is indicated for patients with human epidermal growth factor receptor 2 positive (HER2+) breast cancer, a phase 1 study to evaluate tolerability/safety of eribulin mesylate with trastuzumab has not been conducted. Therefore, a study of eribulin mesylate in combination with trastuzumab was conducted to evaluate dose limiting toxicity (DLT), tolerability/safety, pharmacokinetics (PK), and efficacy and to estimate the recommended dose in Japanese patients with advanced or recurrent HER2+ breast cancer. Eribulin mesylate (1.4 mg/m^2^) was administered on days 1 and 8 of every 3 week cycle. Trastuzumab was administered with a 4 mg/kg loading dose followed by 2 mg/kg weekly doses or with an 8 mg/kg loading dose followed by 6 mg/kg tri-weekly doses. A total of 12 patients (six for each regimen) received eribulin mesylate and trastuzumab. No DLT was observed and the recommended dose of eribulin mesylate in combination with trastuzumab was estimated as 1.4 mg/m^2^. Common adverse events were neutropenia, leukopenia, anaemia and alopecia. This combination therapy was well tolerated and the neutropenia observed was manageable. No PK drug-drug interaction between eribulin and trastuzumab was observed. Since a transient ejection fraction decreased was observed in two patients, cardiac function should be routinely assessed in patients receiving the combination therapy of eribulin mesylate with trastuzumab (ClinicalTrials.gov Identifier: NCT01432886).

## Introduction

Overexpression of human epidermal growth factor receptor 2 (HER2) occurs in approximately 25 to 30 % of breast cancers and is associated with a poor prognosis [1, 2]. Trastuzumab is a humanized IgG1 kappa monoclonal antibody that selectively binds with high affinity to the extracellular domain of the HER2 protein. The studies of weekly trastuzumab monotherapy (4 mg/kg loading dose followed by 2 mg/kg weekly) and combination with paclitaxel was active, well tolerated and prolonged survival in patients with HER2+ metastatic breast cancer [3–5]. Also, comparison of tri-weekly trastuzumab (8 mg/kg loading dose followed by 6 mg/kg tri-weekly dose) with weekly trastuzumab has shown comparable results in both monotherapy and combination therapy with paclitaxel [6, 7]. The combination therapy of a tubulin-targeted drug and trastuzumab appeared to have a superior antitumor effect and a well-tolerated safety profile in the treatment of HER2 + breast cancer [5, 7–9].

Eribulin mesylate, a non-taxane microtubule dynamics inhibitor, is a structurally simplified synthetic analog of the marine natural product halichondrin B. The inhibitory effects of eribulin on microtubule dynamics lead to G_2_/M cell-cycle blocks, disruption of normal mitotic spindle formation, and prolonged mitotic blockage followed by apoptotic cell death [10, 11].

A phase 1 study has been completed in Japanese patients with solid tumors to evaluate the safety and pharmacokinetics (PK) of eribulin mesylate administration on days 1 and 8 of a 21-day cycle. Dose-limiting toxicities (DLTs) were neutropenia/febrile neutropenia, and the recommended dose of eribulin mesylate was determined as 1.4 mg/m^2^ [12]. Based on the results of this phase 1 study in Japan, a phase 2 study was conducted to evaluate the efficacy and safety of eribulin mesylate in patients with locally advanced or metastatic breast cancer previously treated with anthracycline and taxane. Eribulin mesylate demonstrated antitumor activity with an objective response rate (ORR) of 21.3 % (17/80 patients) and a manageable safety profile [13]. This study supported the previous phase 2 study of eribulin mesylate that demonstrated its antitumor activity and safety profile in extensively pretreated breast cancer patients [14, 15].

In a randomized phase 3 study of patients with locally recurrent or metastatic breast cancer who previously received at least two chemotherapeutic regimens including an anthracycline and a taxane, the efficacy and safety of eribulin mesylate (1.4 mg/m^2^, days 1 and 8 of a 21-day cycle) were compared with the treatment of the physician’s choice (TPC). Overall survival (OS) was statistically significantly longer in the eribulin mesylate group than in the TPC group (median OS: 13.1 months vs. 10.6 months, hazard ratio [HR]: 0.81, *p* = 0.041). Furthermore, an updated analysis of OS confirmed the significant increase in OS of the eribulin mesylate group compared with the TPC group (median OS: 13.2 months vs. 10.5 months, HR: 0.81, *p* = 0.014) [16].

Based on these results, the combination therapy of eribulin mesylate and trastuzumab was also expected to provide a superior antitumor effect and favorable safety profile.

Therefore, a phase 1 study of eribulin mesylate in combination with trastuzumab in Japanese patients with advanced or recurrent HER2+ breast cancer was carried out.

## Materials and methods

### Study design and treatment

This was a multi-center, open-label phase 1 study of eribulin mesylate with trastuzumab combination therapy in Japanese patients with advanced or recurrent HER2+ breast cancer (NCT01432886). This study consisted of Part 1 (weekly dose of trastuzumab) and Part 2 (tri-weekly dose of trastuzumab) to evaluate DLT, tolerability/safety, efficacy and PK, and to estimate the recommended dose of eribulin mesylate in this combination therapy.

Eribulin mesylate was administered by 2- to 5-minute i.v. injection on days 1 and 8 of a 21-day cycle. The initial dose of eribulin mesylate was 1.4 mg/m^2^ (equivalent to 1.23 mg/m^2^ eribulin expressed as free base) in combination with trastuzumab treatment. Eribulin mesylate was administered on days 1 and 8, if all of the following criteria were met: (1) neutrophil count ≥1.0 × 10^3^/μL, (2) platelet count ≥7.5 × 10^4^/μL, and (3) non-hematologic toxicity ≤grade 2 (except grade 3 nausea, vomiting, and diarrhea controllable with anti-emetic or anti-diarrheal medication and abnormal laboratory parameters not requiring treatment). If the patient did not meet the criteria, the administration of the next dose was delayed. If the patient met the criteria within a 1-week delay, the 2nd administration of the cycle was implemented, and the next cycle had to be initiated no sooner than 2 weeks after the 2nd administration. If the patient did not meet the criteria within a 1-week delay, the 2nd administration was skipped. Dose reduction of eribulin mesylate could be exercised at the discretion of the investigator if the 2nd administration in a cycle was delayed or skipped. If dose reduction was necessary, the 1.4 mg/m^2^ dose of eribulin mesylate was initially reduced to 1.1 mg/m^2^ and then further reduced to 0.7 mg/m^2^.

In Part 1, trastuzumab was administered by i.v. infusion at 4 mg/kg as a loading dose and at 2 mg/kg weekly. In Part 2, trastuzumab was administered by i.v. infusion at 8 mg/kg as a loading dose and at 6 mg/kg tri-weekly. The infusion time of trastuzumab was 90 min or longer at initial administration and could be shortened to 30 min from the 2nd administration and later. Trastuzumab was administered immediately after eribulin mesylate administration when used in the same day. Concomitant use of other medications or treatments was allowed. However, other anti-cancer drugs, investigational drugs and prophylactic administration of granulocyte-colony stimulating factor (G-CSF) were not permitted during the study.

DLT was evaluated in the first cycle, and if DLTs were observed in none or one of the first three patients, an additional three patients were to be added at the same dose level. If none or one of a total of six patients experienced a DLT, the investigated dose level of eribulin mesylate was to be regarded as tolerable with the trastuzumab combination. In the event that two of six patients reported a DLT, the investigators were to obtain written or verbal advice from an Independent Safety Committee on whether to investigate a decreased dose level of eribulin mesylate (1.1 mg/m^2^) as the initial dose. The initial eribulin mesylate dose level was planned to decrease to 1.1 mg/m^2^ if a DLT was reported in three or more patients. Patients were to continue to receive eribulin mesylate until they no longer received clinical benefit, had progressive disease (PD), or experienced unacceptable toxicity.

The protocol was approved by the Institutional Review Board and conducted in accordance with the Declaration of Helsinki.

### Patient eligibility

Key inclusion criteria included: 20–74 years of age; histologically or cytologically confirmed advanced or recurrent breast cancer; HER2+ tumor score of 3+ by immunohistochemistry staining or gene amplification by fluorescence in situ hybridization (FISH); and any of the following, 1) evidence of recurrence during adjuvant chemotherapy with trastuzumab and taxane, 2) evidence of recurrence within 6 months after adjuvant chemotherapy with trastuzumab and taxane or 3) prior chemotherapy including trastuzumab and taxane for advanced or recurrent breast cancer; normal function in major organs; left ventricular ejection fraction (LVEF) ≥60 % by multigated acquisition scan or echocardiogram (B or M mode); and Eastern Cooperative Oncology Group (ECOG) performance status of 0 or 1.

Key exclusion criteria included: brain metastasis accompanied by clinical symptoms or requiring treatment; severe active infection requiring treatment; pleural effusions, ascites or pericardial effusions requiring drainage; pregnancy or breastfeeding; and refusal of supportive therapy by blood transfusion. All patients provided written informed consent prior to any study procedure.

## Assessments

### DLTs

The following toxicities were to be regarded as DLTs if they occurred in the first cycle and their causal relationship with the study treatment could not be ruled out: grade 4 neutropenia persistent for more than 7 days; grade 3 or above febrile neutropenia; grade 4 thrombocytopenia or grade 3 thrombocytopenia requiring blood transfusion; and non-hematologic toxicity ≥grade 3 (unless grade 3 nausea, vomiting and diarrhea was controllable with an anti-emetic and anti-diarrheal medication, or clinical laboratory abnormalities did not require treatment). It would also be regarded as a DLT if the 2nd eribulin mesylate administration per cycle was delayed and the study treatment could not be resumed from day 22 of cycle 1.

### Safety

The demographic and disease characteristics of breast cancer were recorded at baseline and included HER2 status, estrogen receptor status, progesterone receptor status, and prior therapy complications. Safety assessments were made throughout the study. The factors that were assessed include adverse events (AEs), vital signs, bodyweight, 12-lead electrocardiogram, multigated acquisition scan or echocardiogram, concomitant medications, and clinical laboratory values (hematology, blood biochemistry and urinalysis). AEs were assessed on days 1, 8 and 15 of each cycle. AE severity was classified according to the Japanese version of Common Terminology Criteria for Adverse Events v4.0. QT interval corrected for heart rate using Fridericia’s formula (QTcF) and LVEF assessment were conducted before the treatment, on day 15 of cycle 1, on day 1 of every 4th cycle, and if clinically indicated.

### PK and biochemical methodology

Blood samples were taken for eribulin PK analysis on days 1 and 8 of the first treatment cycle, pre-dose, end of infusion, 30 min, and 1, 2, 4, 24, 72 and 168 h after drug administration. Plasma concentrations of eribulin, measured as the free-base (i.e. non-mesylate) equivalent, were determined by using the validated liquid chromatography- tandem mass spectrometry method. The lower limit of quantitation was 0.2 ng/mL.

In Part 1, blood samples were taken for trastuzumab PK analysis on day 1 (pre-dose, end of infusion, and 3, 4, 24 and 72 h after drug administration) and day 8 and 15 (pre-dose) of cycle 1, and on day 1 (pre-dose) of cycle 2 and later. In Part 2, blood samples of day 1 (pre-dose and 3, 4, 24, 72, 168 and 336 h after drug administration) of cycle 1 and day 1 (pre-dose) of cycle 2 and later cycles were taken. Serum concentrations of trastuzumab were determined by using the validated enzyme-linked immunosorbent assay method. The lower limit of quantitation was 10 μg/mL.

PK parameters for eribulin and trastuzumab were calculated by a non-compartmental approach by using WinNonlin software version 6.2 (Pharsight Corporation, CA, USA). The calculated parameters included: area under the curve extrapolated to infinity (AUC_(0-inf)_), terminal half-life (t_1/2_), total clearance (CL) and volume of distribution at steady state (V_ss_). The maximum observed plasma concentration (C_max_) and time to C_max_ (t_max_) were directly derived from the data.

### Tumor assessment

Tumor response was assessed by the investigators according to the Response Evaluation Criteria in Solid Tumors (RECIST) version 1.1 [17]. Tumor assessments were performed within 28 days prior to the start of treatment, 6 weeks after the first dose and every 6 weeks thereafter, or sooner, if there was clinical suspicion of disease progression.

## Results

Results are based on data collected until an August 2, 2013, cutoff date.

### Patient characteristics

A total of 12 patients received eribulin mesylate and trastuzumab, 6 in Part 1 and 6 in Part 2. These patients were extensively pretreated with a median of 4.5 (range, 1–14) prior chemotherapy regimens. Patient demographics and baseline characteristics are shown in Table 1.

**Table 1:** Patient demographics and baseline characteristics

Parameter	Part 1 (*N* = 6)	Part 2 (*N* = 6)	Total (*N* = 12)
Age, median (range), years	64.5 (58–72)	47.0 (39–64)	60.0 (39–72)
Race, n (%)			
Japanese	6	6	12 (100.0)
ECOG performance status, n (%)			
0	3	2	5 (41.7)
1	3	4	7 (58.3)
ER + and/or PR + disease, n (%)	2	3	5 (41.7)
Number of prior anti-cancer drug treatment, n (%)^a^			
1	0	1	1 (8.3)
2	0	1	1 (8.3)
3	2	0	2 (16.7)
4	1	1	2 (16.7)
≥5	3	3	6 (50.0)
Median (range)	5.0 (3–14)	4.5 (1–6)	4.5 (1–14)
Prior trastuzumab treatment, n (%)	6	6	12 (100.0)
Number of prior trastuzumab treatment, n (%)^a^			
1	2	1	3 (25.0)
2	1	3	4 (33.3)
≥3	3	2	5 (41.7)
Median (range)	2.5 (1–5)	2.0 (1–4)	2.0 (1–5)
Prior taxane treatment, n (%)	6	6	12 (100.0)
Number of prior taxane treatment, n (%)^a^			
1	3	4	7 (58.3)
2	2	2	4 (33.3)
3	1	0	1 (8.3)
Prior anthracycline treatment, n (%)	3	3	6 (50.0)

*ECOG* Eastern Cooperative Oncology Group, *ER* estrogen receptor, *PR* progesterone receptor

^a^Including the number of neoadjuvant, adjuvant and therapeutic therapy

### Treatment

For the overall population, eribulin mesylate and trastuzumab were administered for a median of 7 (range, 2–23) cycles, with eight patients receiving five or more cycles. The numbers of treatment cycles by patients are shown in Table 2. Treatment was discontinued in seven patients (58.3 %) due to PD, and two patients (16.7 %) withdrew due to AEs. Three patients (25.0 %) continued to receive the study drug treatment at the time of the cutoff date. Dose adjustment (reduction, delay or skip) of eribulin mesylate occurred in ten patients (83.3 %); eight patients (66.7 %) had dose reduction, eight patients (66.7 %) had dose delay, and five patients (41.7 %) had to skip one dose per cycle.

**Table 2:** Numbers of treatment cycles by patients

Number of cycles received, n (%)	Part 1 (*N* = 6)	Part 2 (*N* = 6)	Total (*N* = 12)
1–2	–	1	1 (8.3)
3–4	2	1	3 (25.0)
5–8	–	3^a^	3 (25.0)^a^
9–12	2	–	2 (16.7)
13–16	–	1^a^	1 (8.3)^a^
17–20	–	–	–
≥21	2^a^	–	2 (16.7)^a^

^a^3 patients in total were on treatment as of 2 August 2013

### DLTs

DLTs were evaluated on the first cycle, and none of the 12 patients experienced DLT. In cycle 1, grade 3 or 4 neutropenia that led to a dose delay of the day 8 administration of eribulin mesylate occurred in two patients (16.7 %), and a dose was skipped in two patients (16.7 %). Two patients (16.7 %) received concomitant treatment with G-CSF for grade 4 neutropenia. The median time from day 1 of cycle 1 to the nadir in neutrophil count was 15 days (95 % CI:14, 23), and the median time to recovery from the nadir to ≤grade 2 neutropenia was 8 days (95 % CI: 4, 9). One of six patients (16.7 %) skipped a dose of trastuzumab due to an ejection fraction decreased (grade 2) in Part 1 (weekly dose of trastuzumab).

### Adverse events

**Fig. 1:**
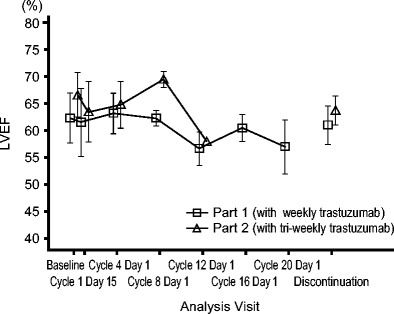
Mean LVEF transition assessed by echocardiogram: Assessment by B mode echocardiogram at the indicated times. Baseline LVEF was ≥60 % by B or M mode echocardiogram in all patients, as defined in the inclusion criteria

The AEs (all grades) experienced by ≥10 % of patients and the total grade 3 or 4 AEs are shown in Table 3. The common AEs were similar in Part 1 and 2. Frequently observed hematologic AEs were neutropenia [100 % (grade 3/4: 100 %)], leukopenia [100 % (grade 3/4: 83 %)] and anaemia [67 % (grade 3/4: 0 %)]. G-CSF was administered to three patients (25.0 %). Frequently observed non-hematologic AEs, which were generally mild and manageable, included alopecia (67 %), pyrexia (42 %), decreased appetite (42 %) and rash (42 %) (all grade 1 or 2). Peripheral neuropathy occurred in four patients (33.3 %), including one patient (8.3 %) with grade 3 neuropathy. The AEs that led to study discontinuation were peripheral neuropathy in one patient (8.3 %) and tumour pain in one patient (8.3 %). An ejection fraction decreased (grade 2) occurred in two patients (16.7 %) (both patients on day 15 of cycle 1 with one patient also in cycle 19), but the patients recovered after 1 week without treatment. The mean LVEF transition is shown in Fig. 1. The other cardiac disorders were second degree atrioventricular block (grade 2) in one patient (8.3 %) and palpitation (grade 1) in two patients (16.7 %); no treatments were required for these adverse advents. No grade 5 AEs or serious AEs were observed.

**Table 3:** Adverse events (All grades in ≥10 % of patients and grades 3/4 in total)

	Part 1		Part 2		Total	
	All grades	Grade 3/4 AEs	All grades	Grade 3/4 AEs	All grades	Grade 3/4 AEs
AE preferred term	*N* = 6	*N* = 6	*N* = 6	*N* = 6	*N* = 12	*N* = 12
Blood and lymphatic system disorders						
Neutropenia	6	6	6	6	12 (100.0)	12 (100.0)
Leukopenia	6	4	6	6	12 (100.0)	10 (83.3)
Anaemia	4	0	4	0	8 (66.7)	0
Lymphopenia	0	0	3	3	3 (25.0)	3 (25.0)
Febrile neutropenia	0	0	1	1	1 (8.3)	1 (8.3)
Cardiac disorders						
Palpitations	2	0	0	0	2 (16.7)	0
Gastrointestinal disorders						
Nausea	1	0	2	0	3 (25.0)	0
Vomiting	0	0	3	0	3 (25.0)	0
Constipation	2	0	0	0	2 (16.7)	0
Stomatitis	1	0	1	0	2 (16.7)	0
General disorders and administration site conditions						
Pyrexia	2	0	3	0	5 (41.7)	0
Malaise	3	0	0	0	3 (25.0)	0
Chest discomfort	1	0	1	0	2 (16.7)	0
Injection site reaction	2	0	0	0	2 (16.7)	0
Infections and infestations						
Lung infection	0	0	2	0	2 (16.7)	0
Tonsillitis	1	0	1	0	2 (16.7)	0
Investigations						
Alanine aminotransferase increased	2	0	1	0	3 (25.0)	0
Aspartate aminotransferase increased	2	0	1	0	3 (25.0)	0
Blood creatine phosphokinase increased	3	0	0	0	3 (25.0)	0
Ejection fraction decreased	1	0	1	0	2 (16.7)	0
Metabolism and nutrition disorders						
Decreased appetite	4	0	1	0	5 (41.7)	0
Hypertriglyceridaemia	1	1	2	2	3 (25.0)	3 (25.0)
Hypophosphataemia	0	0	1	1	1 (8.3)	1 (8.3)
Musculoskeletal and connective tissue disorders						
Myalgia	4	0	0	0	4 (33.3)	0
Muscle spasms	2	0	0	0	2 (16.7)	0
Nervous system disorders						
Dysgeusia	2	0	2	0	4 (33.3)	0
^a^Peripheral neuropathy	4	1	0	0	4 (33.3)	1 (8.3)
Headache	0	0	2	0	2 (16.7)	0
Skin and subcutaneous tissue disorders						
Alopecia	5	–	3	–	8 (66.7)	–
Rash	4	0	1	0	5 (41.7)	0

^a^Peripheral neuropathy includes neuropathy peripheral and peripheral sensory neuropathy in preferred terms

### PK

**Fig. 2:**
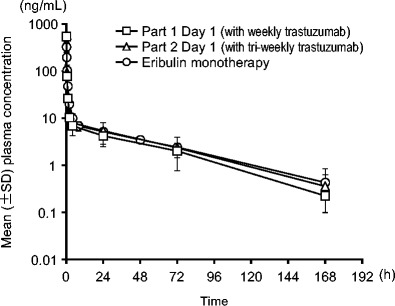
PK analysis of the relationship between mean plasma eribulin concentration versus time profiles for Parts 1 and 2 of the study and for a previous phase 1 study [12] (cycle 1, day 1)

Figure 2 shows the mean plasma eribulin concentration time profile up to 168 h and Table 4 shows the PK parameters of eribulin after eribulin mesylate (1.4 mg/m^2^) administration over 2 to 5 min with trastuzumab on day 1 of cycle 1 in Part 1 and Part 2, as well as previously reported phase 1 study results of eribulin mesylate monotherapy in Japanese patients [12].

**Table 4:** Pharmacokinetic parameters of eribulin (cycle 1, day 1)

PK Parameter (unit)	Part 1 (*n* = 6)	Part 2 (*n* = 6)	Monotherapy (*n* = 6)
C_max_ (ng/mL)	547 ± 128	582 ± 61.0	519.4 ± 107.2
AUC_(0-inf)_ (ng⋅h/mL)	524 ± 137	631 ± 271	672.7 ± 113.7
t_1/2_ (h)	38.1 ± 7.80	35.0 ± 10.8	39.4 ± 8.3
CL (L/h/m^2^)	2.47 ± 0.774	2.12 ± 0.754	1.89 ± 0.33
V_ss_ (L/m^2^)	101 ± 45.3	69.8 ± 11.8	76.3 ± 19.2

Mean ± SD

*AUC*_*(0-inf)*_ area under the concentration-time curve from time zero to infinity, *CL* total clearance, *C*_*max*_ maximum plasma concentration, *t*_*½*_ terminal half-life, *V*_*ss*_ steady-state volume of distribution

In Part 1, the mean values for t_1/2_, CL and V_ss_ after administration of eribulin mesylate on day 1 (Table 4) and Day 8 were 38.1 ± 7.80 and 30.3 ± 3.29 h, 2.47 ± 0.774 and 2.44 ± 0.967 L/h/m^2^, and 101 ± 45.3 and 77.9 ± 37.6 L/ m^2^, respectively. In Part 2, the mean values for t_1/2_, CL and V_ss_ after administration of eribulin mesylate on day 1 (Table 4) and Day 8 were 35.0 ± 10.8 and 31.7 ± 8.58 h, 2.12 ± 0.754 and 1.95 ± 0.721 L/h/m^2^, and 69.8 ± 11.8 and 58.0 ± 6.99 L/m^2^, respectively.

After trastuzumab was administered intravenously in combination with eribulin, trastuzumab was eliminated from the serum biphasically after reaching the C_max_ in both Part 1 and 2. In Part 1, the mean values for t_1/2_, CL and V_ss_ after administration of trastuzumab (4 mg/kg) on day 1 were 115 ± 28.0 h, 0.369 ± 0.0297 mL/h/kg and 62.4 ± 17.9 mL/kg, respectively. In Part 2, the mean values of t_1/2_, CL and V_ss_ after administration of trastuzumab (8 mg/kg) on day 1 were 173 ± 26.7 h, 0.271 ± 0.0343 mL/h/kg and 62.0 ± 4.04 mL/kg, respectively (Table 5).

**Table 5:** Pharmacokinetic parameters of trastuzumab (cycle 1, day 1)

PK parameter (unit)	Part 1 (4 mg/kg) (*n* = 6)	Part 2 (8 mg/kg) (*n* = 6)
C_max_ (μg/mL)	74.9 ± 25.9	194 ± 33.1
AUC (μg⋅h/mL)	10900 ± 868	29800 ± 3740
t_1/2_ (h)	115 ± 28.0	173 ± 26.7
CL (mL/h/kg)	0.369 ± 0.0297	0.271 ± 0.0343
V_ss_ (mL/kg)	62.4 ± 17.9	62.0 ± 4.04

Mean ± SD

*AUC*_*(0-inf)*_ area under the concentration-time curve from time zero to infinity, *CL* total clearance, *C*_*max*_ maximum plasma concentration, *t*_*½*_ terminal half-life, *V*_*ss*_ steady-state volume of distribution

### Antitumor activity

Tumor responses were evaluated by RECIST version 1.1 in all 12 patients. The ORR was 8.3 % (95 % CI: 0.2, 38.5) and tumor responses consisted of a partial response (PR) in one patient (8.3 %), stable disease (SD: including non-complete response (CR)/non-PD) ≥5 weeks) in ten patients (83.3 %) and PD in one patient (8.3 %). The disease control rate (CR + PR + SD ≥11 weeks) was 83.3 % (95 % CI: 51.6, 97.9) and the clinical benefit rate (CR + PR + SD ≥23 weeks) was 50.0 % (95 % CI: 21.1, 78.9) (Table 6).

**Table 6:** Best tumor responses

	Part 1	Part 2	Total
Response category, n (%)	*N* = 6	*N* = 6	*N* = 12
Best tumor response, n (%)			
CR	0	0	0
PR	1 (16.7)	0	1 (8.3)
SD (including non-CR/non-PD)	4 (66.7)	6 (100.0)	10 (83.3)
PD	1 (16.7)	0	1 (8.3)
Not evaluable	0	0	0
Objective response rate	1 (16.7)	0	1 (8.3)
95 % CI	0.4, 64.1	0.0, 45.9	0.2, 38.5
Disease Control Rate	5 (83.3)	5 (83.3)	10 (83.3)
95 % CI	35.9, 99.6	35.9, 99.6	51.6, 97.9
Clinical Benefit Rate	4 (66.7)	2 (33.3)	6 (50.0)
95 % CI	22.3, 95.7	4.3, 77.7	21.1, 78.9

*CI* confidence interval, *CR* complete response, *PR* partial response, *SD* stable disease, *PD* progressive disease

## Discussion

This phase 1 study established the recommended dose of eribulin mesylate as 1.4 mg/m^2^ when administered on days 1 and 8 of a 21-day cycle with appropriate dose adjustment in combination with either weekly trastuzumab (4 mg/kg loading dose, 2 mg/kg/weekly) or tri-weekly trastuzumab (8 mg/kg loading dose, 6 mg/kg/tri-weekly) in Japanese patients with advanced or recurrent HER2+ breast cancer. Eribulin mesylate was suggested to be safe and tolerable in combination with trastuzumab with the same recommended dose as monotherapy [12].

There were no DLTs, grade 5 AEs or serious AEs in this study. The most common AEs of grade 3 or 4 reported in this study were neutropenia and leukopenia, which were hematologic toxicities also found in prior clinical studies of eribulin mesylate monotherapy in Japanese patients [12, 13]. All 12 patients experienced neutropenia, but all events were reversible and easily managed with appropriate dose reduction or a skipped dose. The median time of 8 days required for resolution from nadir in neutrophil count to ≤grade 2 neutropenia in cycle 1 and the low requirement for G-CSF use (25 %) supported the safety of the defined eribulin mesylate administration procedure. The non-hematologic AEs were consistent with the known safety profile of eribulin, with only one grade 3 or higher AE with clinical symptoms (grade 3 peripheral neuropathy). The low incidence of neuropathy was consistent with previous eribulin mesylate studies and the incidence was also lower when compared with taxane studies [12–16, 18].

A retrospective review of seven phase 2 and phase 3 trastuzumab clinical trials reported that the patients treated with trastuzumab were at an increased risk of cardiac dysfunction. The incidence of cardiac dysfunction was highest in patients receiving concomitant trastuzumab and anthracycline/cyclophosphamide (27 %). The risk was substantially lower in patients receiving paclitaxel and trastuzumab (13 %), trastuzumab alone (3 to 7 %), anthracycline/cyclophosphamide alone (8 %), or paclitaxel alone (1 %) [19]. To evaluate the risk of cardiac dysfunction in patients receiving the combination therapy of eribulin mesylate and trastuzumab, periodic assessments of QTcF and LVEF were conducted. Although an ejection fraction decreased (grade 2) was observed in two patients (16.7 %), these patients recovered after 1 week without treatment. The mean LVEF transition was ≥55 % in either part 1 or 2 of the study (Fig. 1). The other cardiac disorders experienced by patients were second-degree atrioventricular block and palpitation, which did not require treatment. Therefore, a clear increased risk of severe cardiac dysfunction resulting from the addition of eribulin mesylate to trastuzumab was not suggested from this study. Since a few cardiac events were observed, cardiac function should be routinely assessed in patients receiving eribulin mesylate in combination with trastuzumab, which is consistent with the recommendation for patients receiving trastuzumab in other combination therapies. The neutropenia observed was manageable, and the non-hematologic AEs were generally mild. Thus, treatment with eribulin mesylate (1.4 mg/m^2^) on days 1 and 8 of a 21-day cycle with appropriate dose adjustment was regarded as tolerable in combination with trastuzumab (either weekly or tri-weekly) in Japanese patients.

The PK profile of eribulin in combination with trastuzumab was similar between the present study and the previously reported phase 1 study of eribulin mesylate monotherapy in Japanese patients [12], including the mean t_1/2_, volume of distribution, and renal and systemic clearance (Fig. 2, Table 4), and as in those studies, the parameters were consistent between days 1 and 8 of the first cycle. The PK profile of trastuzumab (Table 5) was also similar to that in the previous reported phase 1 study of trastuzumab [20]. Combination therapy of trastuzumab with eribulin mesylate did not appear to change the PK profile of either eribulin or trastuzumab. Therefore, no pharmacokinetic drug-drug interaction between eribulin and trastuzumab was indicated.

Although efficacy was not a primary objective, 8 of 12 patients completed more than 5 cycles of treatment and the clinical benefit rate (CBR) was 50.0 % (95 % CI: 21.1, 78.9), which also supports the long-term efficacy and safety of this combination therapy. Recently, the final results of a phase 2 study of eribulin mesylate (1.4 mg/m^2^) in combination with trastuzumab (8 mg/kg as a loading dose followed by a 6 mg/kg tri-weekly dose) as a first-line therapy for locally recurrent or metastatic HER2+ breast cancer was reported. Those results showed: ORR of 71.2 % (37/52 patients), CBR of 84.6 % (95 % CI: 71.9, 93.1), median progression free survival of 11.6 months (95 % CI: 9.1, 13.9) and an acceptable safety profile. The most common AEs of grade 3 or 4 were neutropenia (38.5 %) with clinical laboratory hematologic abnormalities of neutrophils (55.8 %) and peripheral neuropathy (26.9 %) [21]. The low incidence of severe neutropenia compared with that of the present study (100 %) was considered to be due to the difference of prior chemotherapy regimen numbers. The high incidence of severe peripheral neuropathy compared with that of the present study (8.3 %) was likely due to the prolonged duration of treatment in a first-line setting.

In conclusion, 1.4 mg/m^2^ eribulin mesylate administered on days 1 and 8 of a 21-day cycle in combination with either weekly or tri-weekly trastuzumab was well tolerated in extensively pre-treated Japanese patients with advanced or recurrent HER2+ breast cancer. The safety profile shown in the present study and the reported phase 2 study [21] indicates that further evaluation of eribulin mesylate and trastuzumab combination therapy is warranted.
